# High-Temperature and Acid Resistance of Concrete with Recycled, Desert Sand, and Crumb Rubber Blends

**DOI:** 10.3390/ma18184410

**Published:** 2025-09-22

**Authors:** Mohammad Nadeem Akhtar, Khaldoon A. Bani-Hani, Jan Nisar Akhtar

**Affiliations:** 1 Department of Civil Engineering, Fahad Bin Sultan University, Tabuk 71454, Saudi Arabia; khaldoon@fbsu.edu.sa; 2 Civil Engineering Section, University Polytechnic, Faculty of Engineering & Technology, Aligarh Muslim University, Aligarh 202001, India; jannisarakhtar1977@gmail.com

**Keywords:** sustainable sand, desert sand, crumb rubber, silica fume, high temperature, sulfuric acid

## Abstract

Natural sand extraction for concrete manufacturing is a global issue for ecological balance and environmental concerns. This study introduced three mixes with three newly developed sand types to replace natural sand in concrete manufacturing. Additionally, three more mixes were made by incorporating optimized 10% silica fume. The durability of the prepared mixes was evaluated at high temperatures of (150–750 °C) at the interval of 150 °C and against immersion in a 5% sulfuric acid solution for 28, 56, 91, and 182 days, respectively. The study’s results reported the stability of the samples up to 300 °C, and then the fall of the samples started at 450 °C. Severe damage in the samples was formed at about 600 °C, and finally, a total collapse was seen at 750 °C. From (150 to 750 °C), the mix TYPE-3SSFC with a sustainable sand combination (50% recycled sand + 45% desert sand + 5% crumb rubber) and 10% silica fume showed better resistance than the other mixes. The compressive strength in the mix TYPE-3SSFC was 20.6%, 16.3%, 14.7%, 21.3%, 26.5%, and 43.2% higher than the mix TYPE-3SC with 10% silica fume. The mix TYPE-3SSFC with optimized 10% silica fume content showed better resistance against 5% sulfuric acid solution than those without silica fume. By morphological analysis, the mix TYPE-3SSFC showed that the interface improved due to the dense interconnectivity of the concrete mix between the crumb rubber paste and silica fume content. A dense calcite crystal was also seen in the mixture, which confirmed the study’s results. The mix with TYPE 2-Sand (100% recycled sand) revealed inferior results, low stability, and high damage. Thus, 100% recycled sand is not recommended for structural concrete.

## 1. Introduction

On a global basis, the amount of solid trash generated each year keeps rising. According to the estimated data, China alone produces about 1.6–2 billion tonnes of construction and demolition waste (CDW) annually. The construction sector is one of the largest waste generators in the European Union (EU), generating approximately 900 million tonnes [1,2], and in the USA and Japan, about 400 and 80 million tonnes annually [3,4]. It was proposed that an estimated 70% of the CDW generated was to be recycled [2,5]. Among the top 10 countries in the world for CDW generation per capita is the Gulf Cooperation Council (GCC) [6,7]. The GCC country, Saudi Arabia, is experiencing rapid economic expansion and exponential population growth. The infrastructure is growing by demolishing old structures and constructing new cities and industries. For this reason, an exponential increment in CDW was seen in the last decade, continuously increasing yearly [8,9,10]. The alarming rise of CDW-related issues in Saudi Arabia necessitates prompt remedial action. For example, Jeddah, the country’s second largest city, produces 8 million tonnes of CDW annually, and other Saudi Arabian cities like Riyadh, Dammam, and the Tabuk region are also affected [11]. The CDW is a problem that requires serious consideration. It could lead to environmental problems, public discomfort, and land use [12,13,14].

The countries with a central river system, such as India, China, Bangladesh, Egypt, Sudan, Brazil, Russia, and the United States, face serious problems with excessive river sand mining for construction activities, especially when using river sand in concrete manufacturing. According to estimates, river sand utilization in the construction industry amounts to more than 50 billion tonnes annually. Several adverse effects are noticed by the fast removal of river sand for the construction industry, such as disturbing river ecosystems, harming aquatic life, reducing water levels, and affecting the people living on the riverbank, which are serious environmental concerns [15,16,17]. Many other technical issues raised by mining the river sand include river bed scours, which destroy the bridges and structures built in the rivers and along the river bank [16,18]. High-quality river sand is the perfect fine aggregate for manufacturing durable concrete. For this reason, many countries have access to river sand, exporting it to many countries where it is unavailable, and sealing river sand is a million-dollar market [15,18,19,20]. So, it is a serious issue, and excessive river sand mining can be addressed by using alternative available materials to replace river sand in the concrete industry without affecting the engineering properties.

Usually, the recycled aggregate from demolished concrete has lower quality and density. Compared to natural aggregates, recycled aggregate has a higher water absorption value. Therefore, the concrete prepared by incorporating the recycled aggregate has to fulfill the engineering properties, and the mix design is required to obtain the required design and target strength [21,22]. Recent studies ascertain that partially adding recycled sand, crumb rubber, and marble waste to new concrete does not impact its mechanical properties [23,24,25,26]. The natural fine aggregate was substituted by recycled sand at the replacement ratios of 20, 50, and 100% in the study by Vieira et al. [27]. Another study, by Carro et al., incorporated replacement ratios of 25, 50, and 100% [28]. The replacement of natural sand with recycled sand in new concrete at ratios of 25, 50, 75, and 100% was studied by Fernández et al. [29]. The published studies found that the replacement level of up to 25% by an equal volume of natural sand showed the same mechanical properties. When the replacement ratio reached 50%, slight variations in strength were noticed, and at 100%, a significant reduction was observed. One pattern is seen in all the studies, where no unique methodology was used to improve the quality of recycled sand. Most of the studies used the recycled sand percentages directly without any treatment. Recycled sand has high water absorption, which was the main reason for declining mechanical strength at a higher replacement ratio. Vacant desert sand and crumb rubber addition to the recycled sand could be the option to develop more sustainable sand for concrete manufacturing without affecting the strength and durability.

When using or replacing waste resources as sustainable building materials, the most important considerations are safety and the material’s usable life. Concrete’s durability depends on its capacity to withstand any condition, particularly intense heat and chemical attack. Due to their immediate and long-term devastation, fire and chemical attack pose a serious risk to buildings and human life. The experimental study tested the design mix concrete at varying elevated temperatures of 25, 250, 500, and 750 °C. The study’s results showed that recycled sand concrete achieved lower strength than natural sand concrete at elevated temperatures, and at 500 °C, the strength was reduced significantly. When the temperature reached 750 °C, the samples were found to be completely damaged, as studied by [30]. Another study was conducted in experimental research on the temperature range (25–600 °C). It has also been shown that concrete made of recycled sand has lower stability than natural sand concrete at elevated temperatures, and the second pattern was also typical, with the rising temperature lowering the strength. At 400 °C, almost all the strength of the samples made from recycled sand was gone, and complete damage was seen, as shown in [31]. Recycled sand with crumb rubber and steel fiber in new concrete was studied and exposed to high temperatures up to 600 °C. However, increasing the crumb rubber percentages decreased the strength at standard temperature. At higher temperatures, adding crumb rubber resulted in stability in the samples and better performance than those without crumb rubber. This could occur due to crumb rubber’s ability to absorb heat [32]. In the case of using crumb rubber in design mix concrete, the primary challenge is the crumb rubber’s thermal decomposition, which ultimately results in a reduction in strength. The thermal decomposition of the crumb rubber particles was confirmed when the temperature reached above 250 °C due to the release of volatile gases, ultimately increasing the risk of spalling. Using the fibers with suitable additives reduced the internal pressure and helped to increase the spalling resistance. Past studies have reported that the increasing crumb rubber content has reduced compressive strength. This reduction in strength is directly proportional to the crumb rubber percentages. For this reason, the optimal percentage of crumb rubber in each design mix should be decided upon to overcome this issue. Furthermore, using suitable admixtures and additives with crumb rubber content makes it suitable for use in the design mix concrete to contribute to a positive outcome, as studied by [33,34,35].

The published studies have disclosed that recycled coarse aggregate can be used in making new concrete. However, sustainable fine aggregate (sand) in new concrete is limited. In addition, the durability of the sustainable sand concrete was not addressed extensively. Furthermore, few experimental investigations have been conducted on sustainable sand concrete performance against high temperatures and sulfuric acid solution. The objective of this study is to assess the durability of sustainable sand concrete against elevated temperatures, sulfuric acid attack, and the microstructural characteristics of developed concrete. The present study evaluates the durability characteristics of modified sustainable sand concrete samples exposed to high temperatures and immersion in sulfuric acid solution. The modified sand was TYPE 1-Sand (100% Manufactured sand) and TYPE 2-Sand (100% recycled sand). The third, TYPE 3-Sand, was developed by utilizing the prepared recycled sand (RS) and desert sand (DS), along with the crumb rubber (CR) percentages (50% RS + 45% DS + 5% CR), respectively. The performance of concrete prepared by adding TYPE 1, 2, and 3 sand combinations was evaluated by compressive strength, residual compressive strength, mass loss, and the morphological characteristics of damaged samples.

## 2. Experimental Methodologies of the Study

### 2.1. Preparation Methodology of Different Sand Types

In this study, three types of fine aggregate were developed using four types of sand: manufactured sand (MS), recycled sand (RS), desert sand (DS), and crumb rubber (CR), respectively. The DS and CR were directly collected from the Abin Construction Company, Tabuk, Saudi Arabia. The manufactured sand (MS) was collected from the crushing plant situated at the construction site of Abin Construction Company, Tabuk, Saudi Arabia. For recycled sand (RS), the end-of-life (EOL) concrete was taken from different demolished sites and crushed in the sand fraction (≤5 mm) at the crushing plant of Abin Construction Company, Tabuk, Saudi Arabia. The prepared sand was washed, cleaned, and sieved in Fahad Bin Sultan University’s materials lab, Saudi Arabia.

The manufactured sand (MS) and recycled sand (RS) were utilized to develop TYPE 1-Sand and TYPE 2-Sand, respectively. After several trial observations, the MS and RS particles were separated into three types of fractions (4.75–2.36 mm), (2.36–1.18 mm), and (1.18–0.075 mm), as shown in Table 1. This technique ensures perfect gradation, maintains the fineness modulus (FM), and, most importantly, avoids fine dust particles below 0.075 mm in the developed design mix concrete for this study of TYPE 1-Sand and TYPE 2-Sand. The correct percentages of each fraction are also a matter of concern. Several trials optimized the correct percentage of each fraction to obtain the best combination of MS and RS properties of the sand in concrete, as suggested by [36]. Finally, the best composition was obtained, as mentioned in Table 1. Desert sand (DS) was introduced to develop a sustainable TYPE 3-Sand combination by adding prepared recycled sand (RS) and crumb rubber (CR) fractions. The DS particles are fine and fail to fulfill the requirement of sand utilization in concrete, as suggested by the [36] code of practice. This study suggests a novel approach to developing green sand by optimizing the fine DS by adding RS and CR in the fraction mentioned in Table 1. After utilizing different fraction combinations, a final combination of (50% recycled sand + 45% desert sand + 5% crumb rubber) achieved the desired physical properties to meet the requirement set by [36]. Table 1 shows all the fractions and percentages of the developed sand combinations.

### 2.2. Engineering Properties of Prepared Sand Combinations

The engineering properties of the developed sand combination, such as particle size distribution by gradation curves, and the physical and chemical properties of the green sand combination, such as specific gravity and water absorption, sand equivalent value, pH, sulphate, TDS, and chloride, were reported, as mentioned in Table 2.

#### 2.2.1. Gradation Curves of Prepared Sand Combinations

**Figure 1:**
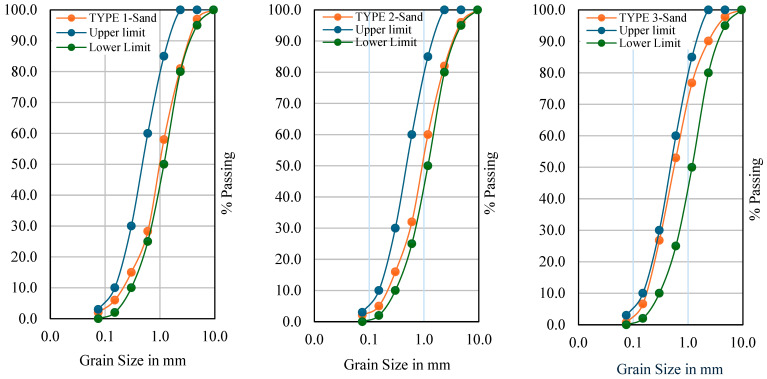
Particle size distribution curves of developed sand Types 1, 2, and 3.

Figure 1 reports the particle size distribution curves of the three sand TYPES 1, 2, and 3 with the standard limits of gradation set by [43]. TYPE 1-Sand with (100% manufactured sand) showed coarser particles. It can be seen from Figure 1 that TYPE 1-Sand is close to the lower limits. However, TYPE 1-Sand satisfied the requirement of sand utilization for concrete as the curve lies between the upper and lower bounds [43]. Figure 1 also shows that TYPE 2-Sand (100% recycled sand) is slightly closer to the lower limit than TYPE 1-Sand. It can be seen from Figure 1 that TYPE 1-Sand and TYPE 2-Sand were found to have almost similar curves. This is because the preparation methodology for both types of sand was the same.

Figure 1 also reports the gradation curve of TYPE 3-Sand with the combination (50% recycled sand + 45% desert sand + 5% crumb rubber). TYPE 3-Sand was found to be closer to an upper limit, as shown in Figure 1. This is due to the higher percentages of fine particles in TYPE 3-Sand. TYPE 3-Sand falls within the standard limits. Hence, TYPE 1-Sand, TYPE 2-Sand, and TYPE 3-Sand all fall within the standard limits and are suitable for utilization in design mix concrete.

#### 2.2.2. Physical Parameters of Concrete Mix Design

In this study, the standard ACI-211.1-91 [36,37,38,44] was utilized to evaluate the design mix proportions. A detailed description of all physical parameters with the standard specification is given in Table 2. Several methods were used to ensure that the physical material properties met specific requirements, as shown in Table 2. Specific gravity values are essential in calculating the fine aggregate mass for the concrete design mix, and absorption is required to evaluate the extra water. The sand equivalent values of the developed sand types were also measured. This parameter is measured to identify the sand samples’ clay, fine particles, granulated soil, and dust. Over 75% of the standard values are acceptable for sand utilization in design mix concrete.

### 2.3. Design Mix Ingredients and Sample Preparation

In this study, 6 concrete mixes were developed. TYPE 1-Sand (100% manufactured sand) was utilized to prepare the reference mix and designated by TYPE-1SC*. The second mix was prepared with TYPE 2-Sand (100% recycled sand), scheduled TYPE-2 Sand Concrete (TYPE-2SC). The combination (50%RS + 45%DS + 5%CR) is used to prepare green sand, which is designated TYPE-3 Sand Concrete (TYPE-3SC). Three additional concrete mixes, TYPE-1SSFC, TYPE-2SSFC, and TYPE-3SSFC, were also prepared by adding 10% optimized silica fume in each designated mix TYPE-1SC*, TYPE-2SC, and TYPE-3SC, respectively.

**Figure 2:**
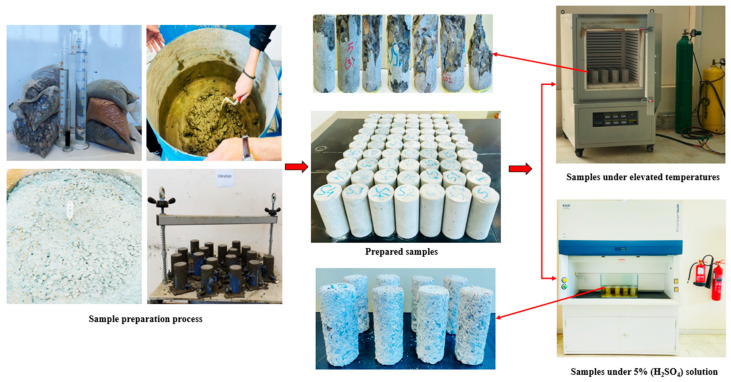
A detailed setup of the lab work for this study.

Table 3 shows the design mix concrete proportions of all 6 developed mixes of this study. The stepwise detailed methodology for the experimental lab program is described in Figure 2. The detailed experimental process of sample preparation to evaluate the deterioration behavior of newly developed sand concrete by elevated temperatures and submergence in a 5% sulfuric acid (H_2_SO_4_) solution is also shown systematically in Figure 2. The cylindrical samples (75 mm diameter × 150 mm of height) were placed in an electric furnace for elevated temperature and a glass chamber for sulfuric acid (H_2_SO_4_) solution. To evaluate each parameter after exposure to high temperatures and 5% sulfuric acid solution, an average of 3 samples from each was utilized to analyze the results.

### 2.4. Muffle Furnace

The equipment of the muffle furnace PT-M1200-216L, from Fahad Bin Sultan University laboratory, Tabuk, Saudi Arabia, was used to examine the performance of concrete specimens at high temperatures, as shown in Figure 2. This furnace uses an electric wire to increase the inside chamber temperature to a maximum capacity of 1200 °C. The heating rate of the chamber can be adjusted between 0 and 10 °C/min with a continuous working temperature of 1100 °C. This study exposed each prepared mix to a temperature range of (150–750 °C). The temperature control program for each temperature range is described in Table 4. The temperature range was designated by (T) in °C and time (t) in min. Each mix was exposed to the required time set in the program, which was 2 h (120 min). The increasing temperature in the program was set to 5 °C per minute, as shown in Table 4. When the cycle of the set program was completed and the temperature reached zero, the furnace was opened for 24 h. This is because of safety guidelines.

## 3. Discussion on the Results of the Study

### 3.1. High Temperature Performance of Newly Developed Concrete

#### 3.1.1. Deterioration of the Samples Under Compressive Strength

The compressive strength of newly developed sand concrete was compared with criteria 1, 2, and 3 selected by [37,38,44]. Criterion 1 stated that the field-cured compressive strength must reach (0.85 *f′c* = 29.75 MPa). Criterion 2 was the selected design strength (*f′c* = 35 MPa) for structural concrete, and finally, the target compressive strength (*f′_cr_* = 43 MPa) was decided by criterion 3. The deterioration behavior of the prepared mixes TYPE-1SC*, TYPE-2SC, TYPE-3SC with the TYPE-1, TYPE-2, and TYPE-3 sand combinations, and the same mixes with 10% silica fume addition TYPE-1SSFC, TYPE-2SSFC, and TYPE-3SSFC, was tested through strength characteristics at ambient temperature (AT) and the elevated temperatures (150–750 °C) at an interval of 150 °C, respectively.

**Figure 3:**
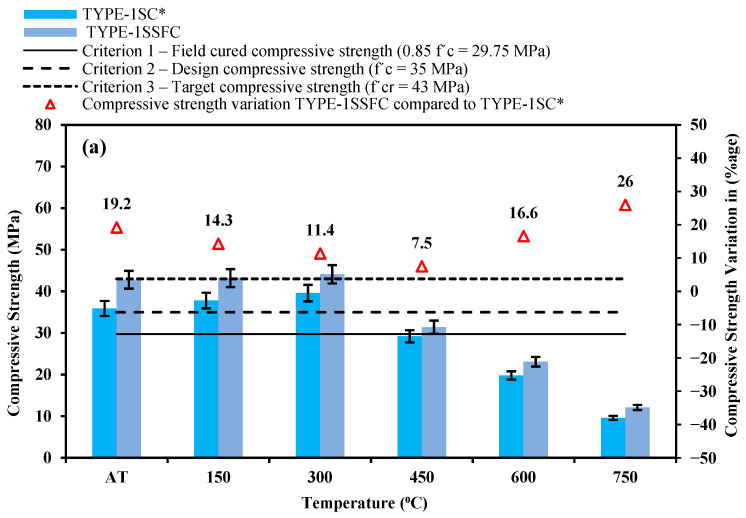

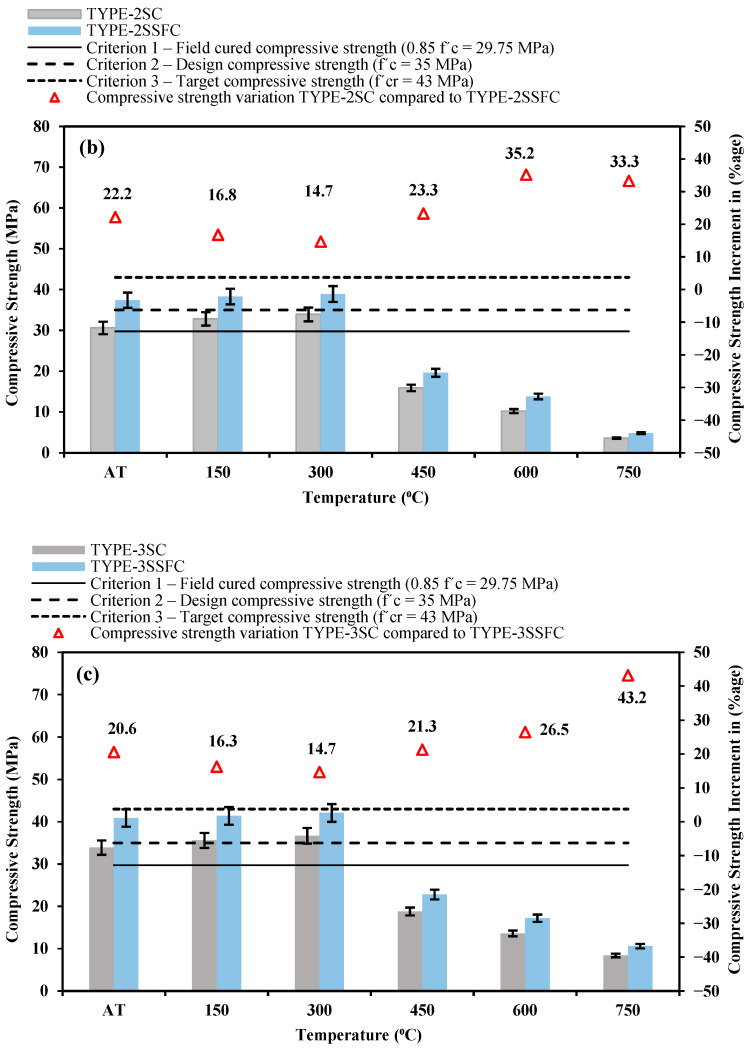
(**a**–**c**) Types 1, 2, and 3 developed sand concrete compressive strength comparison with and without silica fume content.

The mix TYPE-1SC* (100% manufactured sand) and the mix TYPE-1SSFC with optimized 10% silica fume were compared; this is reported in Figure 3a. The compressive strength at the standard room temperature for the mixes TYPE-1SC* and TYPE-1SSFC was observed at 35.9 and 42.8 MPa. It can be seen from Figure 3a that the addition of silica fume in the mix TYPE-1SSFC showed an increment in the compressive strength, and criteria 1 and 2 successfully passed and almost reached criterion 3 (*f′_cr_* = 43 MPa). The compressive strengths in the control mix TYPE-1SC* without silica fume at (150–750 °C) were reported to be 37.8, 39.6, 29.2, 19.8, and 9.6 MPa, respectively. In the mix, TYPE-1SSFC without 10% silica fume at (150–750 °C) was reported to be 43.2, 44.1, 31.4, 23.1, and 12.1 MPa. In the mix, TYPE-2SSFC with 10% silica fume with respect to (w.r.t.) mix TYPE-2SC without silica fume was recorded at 19.2%, 14.3%, 11.4%, 7.5%, 16.6%, and 26%, respectively.

Figure 3b compares the mixes TYPE-2SC (100% recycled sand) and TYPE-2SSFC with silica fume content of 10%. The mixes TYPE-2SC and TYPE-2SSFC at room temperature were observed at 30.6 and 37.4 MPa. Figure 3b shows that adding silica fume in the mix TYPE-2SSFC showed an increment in the compressive strength, and criteria 1 and 2 successfully passed and failed criterion 3 (*f′_cr_* = 43 MPa). The compressive strengths in the control mix TYPE-2SC without silica fume (150–750 °C) were 32.8, 33.9, 15.9, 10.2, and 3.6 MPa. The compressive strengths in the control mix TYPE-2SSFC without 10% silica fume at (150–750 °C) were reported to be 38.3, 38.9, 19.6, 13.8, and 4.8 MPa. The increment in the mix TYPE-2SSFC with respect to (w.r.t.) mix TYPE-2SC without silica fume was recorded at 22.2%, 16.8%, 14.7%, 23.3%, 35.2%, and 33.3%, respectively.

The mixes TYPE-3SC (50% recycled sand + 45% desert sand + 5% crumb rubber) and TYPE-3SSFC with 10% optimized silica fume were compared, as reported in Figure 3c. At the standard room temperature, the compressive strength of the mixes TYPE-3SC and TYPE-3SSFC was observed at 33.9 and 40.9 MPa. The mix TYPE-3SSFC showed an increment in the compressive strength, and criteria 1 and 2 successfully passed and almost reached criterion 3 (*f′_cr_* = 43 MPa). The compressive strengths in the control mix TYPE-3SC* without silica fume at (150–750 °C) were reported to be 35.6, 36.7, 18.8, 13.6, and 8.4 MPa. In the mix, TYPE-3SSFC without 10% silica fume at (150–750 °C) was reported to be 41.4, 42.1, 22.8, 17.2, and 10.6 MPa, respectively. The compressive strength increment in the mix TYPE-3SSFC with 10% silica fume with respect to (w.r.t.) mix TYPE-3SC without silica fume was recorded at 20.6%, 16.3%, 14.7%, 21.3%, 26.5%, and 43.2%.

Figure 3a–c show the compressive strength results of the study. Some patterns were observed in the experimental results. It is seen from the results that newly developed sand concrete TYPE-1SSFC, TYPE-2SSFC, and TYPE-3SSFC with the optimized 10% silica fume performed well at each heating stage (150–750 °C) compared to the mixes TYPE-1SC*, TYPE-2SC, and TYPE-3SC. The silica fumes with developed sand help resist temperature, and less deterioration was reported in the samples. Furthermore, it is also pointed out that the design mix TYPE-3SSFC (50% recycled sand + 45% desert sand + 5% crumb rubber), along with 10% silica fume, performed better than the mix TYPE-2SSFC (100% recycled sand) without silica fume. The rate of strength change at each temperature range in the mix TYPE-3SSFC is less than that of TYPE-2SSFC. This could be because the crumb rubber has high thermal resistance, which was supported by a past published study [45].

#### 3.1.2. Deterioration of the Samples Under Residual Compressive Strength

**Figure 4:**
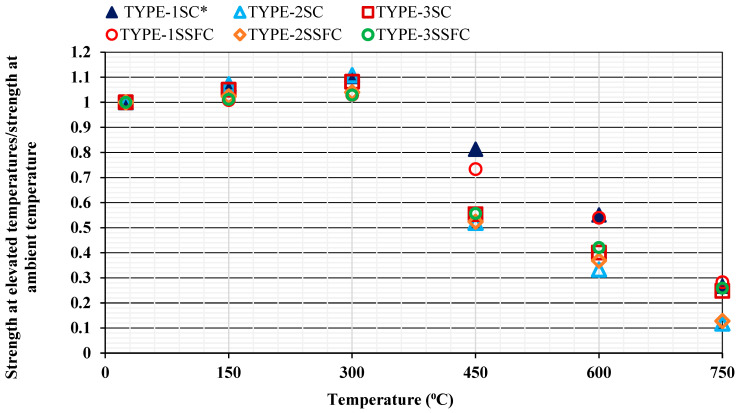
Strength comparison at high temperatures with the standard room temperature.

The estimation of the ratio of the compressive strength at elevated temperatures (150–750 °C) w.r.t standard room temperature 25 °C, is shown in Figure 4. In all mixes, a clear trend was observed that the increment in the strength values was seen in the starting elevated temperatures of 150 °C and 300 °C. At this temperature range (150–300 °C), the free water available in samples starts evaporating. Because of the evaporation of the remaining pores, the concrete mix starts reducing its size. Ultimately, this phenomenon helps to increase the compressive strength at this temperature range (150–300 °C), as shown in Figure 4. As reported earlier in the materials properties, the maximum amount of water absorption obtained in the TYPE 2-Sand (100% recycled sand) is why more evaporation was found in the mix TYPE-2SC. More or less, however, this instance was found in all the mixes, as shown in Figure 4. Another reason for the increasing strength with temperature is the packing of developed sand in the concrete mixture. The morphology of sand TYPE-1, TYPE-2, and TYPE-3 differs, such as in the size and shapes of aggregate distribution, affecting the hardened concrete wet density and particle packing. Using different sand combinations in mixes could reveal several reasons for this phenomenon. Furthermore, replacing 10% silica fume with OPC produced additional C-S-H gel, and the decomposition did not start up to 300 °C. That is why all the mixes showed the same pattern for raising the strength up to 300 °C in all the samples.

The strength continues to reduce between 450 °C and 600 °C. In all the prepared mixes, sudden strength reduction was compared to the strength at standard room temperature at 25 °C. The mix TYPE-1SC* with all-natural ingredients and mix TYPE-1SSFC, when 10% OPC was replaced with silica fume, performed better than other mixes, and the reductions were only about 20% and 27%, respectively. Other mixes reported a 40–50% reduction. At 600 °C, more reduction was reported with a similar pattern. TYPE-2SC and TYPE-2SSFC with (100% recycled sand) reported the maximum reduction in the mixes. In the recycled sand mixture, the outer face of the samples was weakened because of the interfacial transition zone (ITZ). Ultimately, more cracks were formed at the early loading. It was continued till the ultimate failure load was reached in the samples. The early formation of cracks helps in reaching early failure, and it was the cause of the reduction in strength.

The mixes TYPE-3SC and TYPE-3SSFC with the sand combination (50% recycled sand + 45% desert sand + 5% crumb rubber) were similar to the reference mix. The crumb rubber resistance against heat was better than that of the samples without crumb rubber, which further caused resistance to concrete breaking. When the temperature reaches above 600 °C, the mixes formed with crumb rubber retain some residual strength, preventing full damage to the samples. However, the correct percentage of crumb rubber is a matter of concern; otherwise, it weakens the paste and could further reduce its compressive strength. Therefore, an optimized rubber content is recommended for newly developed concrete against higher temperatures. This point of view is supported by a published study [32,33,34,35].

Another important fact is that between 450 °C and 600 °C, the degradation of some siliceous aggregates was found at their peak. As seen in Equation (1), the dissection of portlandite was the primary reason for decreasing the significant strength in this temperature range (450–600 °C).

(1) $${\mathrm{Ca}(\mathrm{OH})}_{2\;}\to \;\mathrm{CaO}+H_{2}O\;↑$$

At 750 °C, most samples were damaged, and the strength evaluation was quite difficult. Only a few samples were found to be stable, those that did not fully collapse. The samples with (crumb rubber + silica fume) content were observed to have better stability than other mixes. The samples with (100% recycled sand) at 750 °C were almost completely damaged. The secondary C-S-H gel formation lost moisture above 300 °C, and the final breakdown between 600 °C and 750 °C was responsible for the complete damage of the samples. The thermo-chemical spalling process compromises the structure. Past studies supported this point of view [7,46,47,48,49,50,51] and reported similar discussions.

### 3.2. Performance of the Developed Sand Concrete Against Sulfuric Acid

#### 3.2.1. Sulfuric Acid Attack on Prepared Mixes of This Study

Concrete’s acid reactivity must be examined in any study of the material’s durability characteristics. It is necessary to assess concrete’s resistance to acid attacks. Prolonged chemical exposure during the concrete-making process might result in acid corrosion. Rain also contains acids due to air pollution. Rain causes chemical processes that raise the quantities of sulfur dioxide and nitrogen dioxide in the atmosphere and make it more acidic. Carboxylic, sulfuric, and nitric acids make up the majority of rain. The hardened concrete needs to be tested in various acidic solutions to evaluate its resistance to acidic solutions. The present study reported the deterioration of samples against an acid attack (5% H_2_SO_4_ solution).

A glass jar was used to evaluate the experimental program to keep the samples at 28, 56, 91, and 182 days in a (5% H_2_SO_4_ solution). The purity of sulfuric acid was 97%, and the solution was prepared accordingly. The sulfuric acid solution was agitated 2–3 times per week. The solution pH was measured by a pH meter. After 28 days of the standard curing period of normal water, the samples were transferred to the glass jar with (5% H_2_SO_4_ solution) for 28, 56, 91, and 182 days of deterioration of the samples against 5% sulfuric acid solution. After completing the duration of each 28, 56, 91, and 182 days, the samples were tested for deterioration against a (5% H_2_SO_4_ solution).

#### 3.2.2. Deterioration of Hardened Concrete by Mass

**Figure 5:**
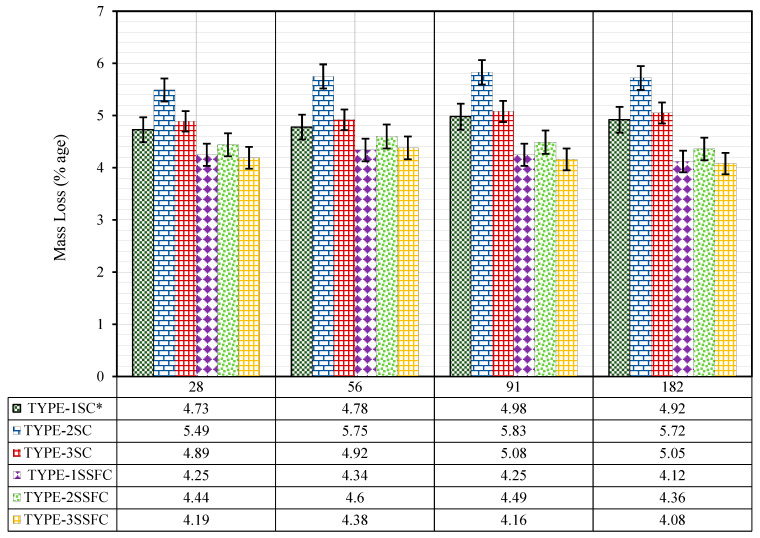
Mass loss of the hardened concrete in a (5% H_2_SO_4_ solution).

The mass of each mix of the TYPE-1SC*, TYPE-2SC, TYPE-3SC, TYPE-1SSFC, TYPE-2SSFC, and TYPE-3SSFC samples was taken out from the (5% H_2_SO_4_ solution) and measured by Equation (2) after each duration of 28, 56, 91, and 182 days. After each duration, time was taken by σ_2,_ and before immersion, denoted by σ_1._ The average change in mass of three samples of each mix of TYPE-1SC*, TYPE-2SC, TYPE-3SC, TYPE-1SSFC, TYPE-2SSFC, and TYPE-3SSFC after 28, 56, 91, and 182 days in (5% H_2_SO_4_ solution) is reported in Figure 5.

(2) $$\mathrm{Mass}\;\mathrm{loss}:\%=\frac{m_{1}-m_{2}}{m_{1}}\;\times \;100$$

The mass loss in the reference mix TYPE-1SC* (100% manufactured sand) and the mix TYPE-1SSFC with 10% optimized silica fume in the reference mix at 28, 56, 91, and 182 days was reported (4.73, 4.78, 4.98, and 4.92) and (4.25, 4.34, 4.25, and 4.12), respectively. Critical observations were obtained from the results of the study. Firstly, the maximum deterioration of the samples against a (5% H_2_SO_4_ solution) was seen within the first 28 days of immersion, and later on, the rate of deterioration slowed. Secondly, the deterioration was neutralized at 182 days of immersion and did not show further reduction. Finally, it has also been observed that the same mix TYPE-1SSFC with the addition of silica fume has a better resistance against (5% H_2_SO_4_ solution) than the mix without silica fume TYPE-1SC*.

The mass loss in the mix TYPE-2SC (100% recycled sand) and TYPE-2SSFC with 10% optimized silica fume in the mix TYPE-2SC at 28, 56, 91, and 182 days was reported (5.49, 5.75, 5.83, and 5.72) and (4.44, 4.46, 4.49, and 4.36), respectively. It was observed that the pattern of the results is almost similar to the reference mix discussed above. However, the deterioration in samples against a (5% H_2_SO_4_ solution) is more than the reference mix. The deterioration was neutralized at 182 days of immersion, similar to the reference mix. It was also observed that the same mix, TYPE-2SSFC, with the addition of 10% silica fume, has a much better resistance against (5% H_2_SO_4_ solution) than the reference mix.

The mass loss in the reference mix TYPE-3SC (50% recycled sand + 45% desert sand + 5% crumb rubber) and the mix TYPE-3SSFC with 10% optimized silica fume in the mix TYPE-3SC at 28, 56, 91, and 182 days was reported (4.89, 4.92, 5.08, and 5.05) and (4.19, 4.38, 4.16, and 4.08), respectively. The results showed that deterioration in the mix TYPE-3SC and TYPE-3SSFC with and without silica fume was almost similar to the reference mix and was found to be better than that with TYPE-2SC (100% recycled sand). However, the pattern of the results was the same in all the mixes: the maximum deterioration of samples against a (5% H_2_SO_4_ solution) was seen within the first 28 days of immersion, and the rate of deterioration slowed. Secondly, the deterioration was neutralized at 182 days of immersion and did not show further reduction. Finally, it was also observed that the mix with 10% silica fume has a better resistance against 5% sulfuric acid solution than the mix without silica fume.

It was concluded from the analysis of the results that the maximum deterioration was reported in the first 28 days when the samples were immersed in a (5% H_2_SO_4_ solution). The chemical reaction in the early days was fast because H_2_SO_4_ reacted with Ca(OH)_2_, gypsum precipitated in the solution, and the particles from the samples were removed, which was the reason for the reduction in the samples’ mass. This pattern was similar in all the mixes up to 28 days of immersion. However, maximum deterioration was seen in the TYPE-2SC mix (100% recycled sand). This is due to the presence of leftover cement layers in (100% recycled sand) that higher precipitation. Ultimately, more mass reductions were reported. As and when the duration was increased beyond 28 days, the variation in pH was reported, and the neutralization reaction slowed down. A slight variation in mass increment was observed 56 and 91 days later. This phenomenon was about to be neutralized when the immersion period reached 182 days. Therefore, a negligible reduction was noticed instead of an increase, as seen in Figure 5.

Another interesting fact was that the optimized 10% silica fume incorporation with developed sand found better resistance against (5% H_2_SO_4_ solution) than those without silica fume. Because silica fume combines with the calcium hydroxide in the samples to form calcium silicate, its addition reduces mass loss in the hardened concrete. When silica fume dosages mix with water to form a silicate, more C-S-H gel is produced. Equation (4) illustrates the reaction between gypsum and calcium aluminate to make ettringite, whereas Equation (3) illustrates gypsum production. Silica fume reacts with calcium hydroxide to form calcium silicate and water, as shown in Equation (5). Equation (6) states that these calcium silicates react with water to produce energy, calcium hydroxide, and hydrated calcium silicate. As seen in Equation (7), silica gel and hydrated calcium sulphate (gypsum) were created when sulfuric acid was introduced to the C-S-H gel from Equation (6).

(3) $${\mathrm{Ca}(\mathrm{OH})}_{2}+H_{2}{\mathrm{SO}}_{4}\;\to \;{\mathrm{CaSO}}_{4}\cdot 2H_{2}O\;$$

(4) $$3\mathrm{CaO}\cdot {\mathrm{Al}}_{2}\cdot 12H_{2}O+3\left({\mathrm{CaSO}}_{4}\cdot 2H_{2}O\right)+14H_{2}O\;\to \;3\mathrm{CaO}\cdot {\mathrm{Al}}_{2}O_{3}\cdot 3{\mathrm{CaSO}}_{4}\cdot 32H_{2}O$$

(5) $${\mathrm{SiO}}_{2}+{\mathrm{Ca}(\mathrm{OH})}_{2}\to \;{\mathrm{CaSiO}}_{3}+H_{2}O$$

(6) $$2{\mathrm{Ca}}_{3}{\mathrm{SiO}}_{5}+7H_{2}O\;\to \;3\mathrm{CaO}\cdot 2{\mathrm{SiO}}_{2}\cdot 4H_{2}O+3{\mathrm{Ca}(\mathrm{OH})}_{2}+\mathrm{Energy}$$

(7) $$3\mathrm{CaO}\cdot {\mathrm{SiO}}_{2}\cdot 3H_{2}O+H_{2}SO_{4}\;\to \;{\mathrm{CaSO}}_{4}\cdot 2H_{2}O+{\mathrm{Si}(\mathrm{OH})}_{4}$$

Furthermore, the fine particles of silica fume, desert sand, and crumb rubber filled the concrete’s micropores, consequently reducing permeability and porosity, thus helping to improve its ability to resist sulfuric acid attack in the mix. In addition, the mix TYPE-3SSFC with a developed sand combination (50% recycled sand + 45% desert sand + 5% crumb rubber) was the best sustainable mix. Due to the fine particles of desert sand, crumb rubber, and silica fume, a cement paste filled the concrete pores of hardened concrete. It helped reduce the porosity of the hardened cement paste, and ultimately, resistance against a (5% H_2_SO_4_ solution) was formed. Since the developed concrete utilized different combinations of waste materials, a direct comparison is impossible. However, the results of the most recent investigations substantially resemble those of the published study [52].

#### 3.2.3. Deterioration of Hardened Concrete by Strength

After measuring the mass of samples of each mix, TYPE-1SC*, TYPE-2SC, TYPE-3SC, TYPE-1SSFC, TYPE-2SSFC, and TYPE-3SSFC, the compressive strength of the same samples was evaluated. The average of three identical samples provided each mix’s compressive strength, and finally, the strength reduction was estimated.

**Figure 6:**
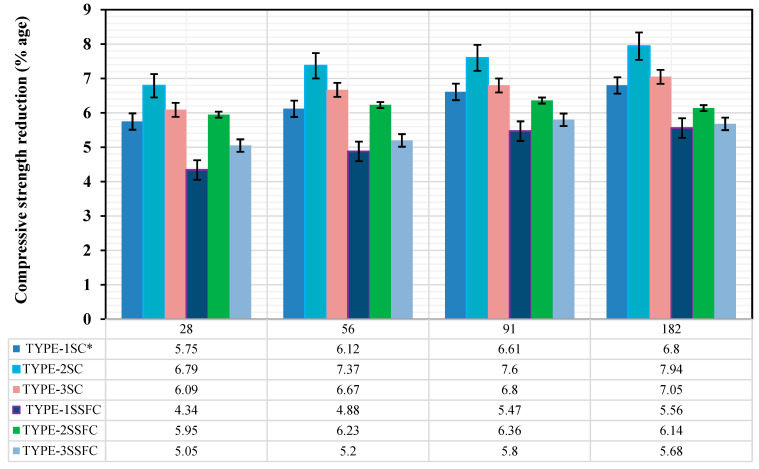
Strength reduction of hardened concrete in a 5% sulfuric acid solution.

The compressive strength reduction of each mix, TYPE-1SC*, TYPE-2SC, and TYPE-3SC without silica fume content, at 28, 56, 91, and 182 days was reported (5.75, 6.12, 6.61, and 6.8), (6.79, 7.37, 7.6, and 7.94), and (6.09, 6.67, 6.8, and 7.05), respectively. The prepared mixes TYPE-1SSFC, TYPE-2SSFC, and TYPE-3SSFC with 10% optimized silica fume at 28, 56, 91, and 182 days were found (4.34, 4.88, 5.47, and 5.56), (5.95, 6.23, 6.36, and 6.14), and (5.05, 5.2, 5.8, and 5.68), as shown in Figure 6.

The lower the mass loss, the greater the compressive strength reduction. It is also clearly shown by the results from Figure 6 that the mixes TYPE-1SSFC, TYPE-2SSFC, and TYPE-3SSFC with 10% silica fume showed less reduction in compressive strength than the mixes TYPE-1SC* TYPE-2SC, and TYPE-3SC without silica fume content. As per the discussion in the above section on mass loss, the exact utilization of silica fumes helped decrease porosity and its occurrence. The mixes with 10% silica fume content showed better resistance to 5% sulfuric acid solution. The developed mix TYPE-3SSFC with the sand combination (50% recycled sand + 45% desert sand + 5% crumb rubber), along with 10% silica fume, showed almost the same performance as the reference mix and developed better resistance to acid attack. However, a direct comparison is challenging because the current study is based on recently produced modified sand concrete. The results of this investigation are consistent with past research that used 10% silica fume as a partial substitute for OPC in concrete to prevent sulfuric acid attacks [53,54].

#### 3.2.4. Correlation Between Mass Loss and Strength Reduction

**Figure 7:**
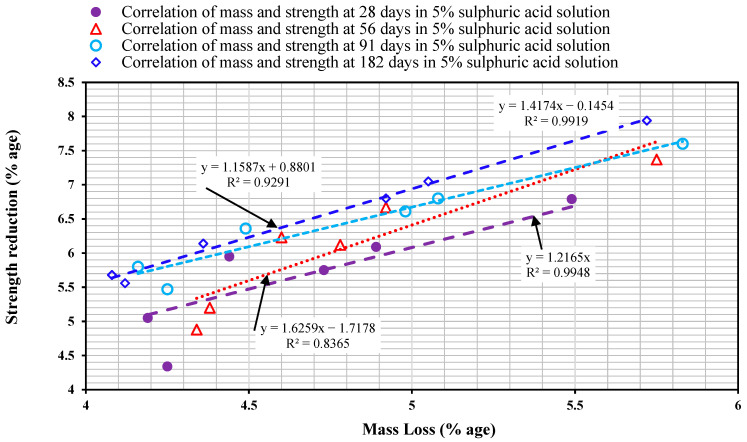
Correlation of mass loss and compressive strength reduction.

Figure 7 shows the correlation of the mixes TYPE-1SC* TYPE-2SC, TYPE-3SC, TYPE-1SSFC, TYPE-2SSFC, and TYPE-3SSFC between mass and compressive strength loss. The correlation in the mixes TYPE-1SC* TYPE-2SC, TYPE-3SC, TYPE-1SSFC, TYPE-2SSFC, and TYPE-3SSFC was examined in (5% H_2_SO_4_ solution) at 28, 56, 91, and 182 days. The mass loss in terms of compressive strength reduction in plain concrete was evaluated as a durability factor against acid attack in sustainable concrete performance.

In the mixes TYPE-1SC* TYPE-2SC, TYPE-3SC, TYPE-1SSFC, TYPE-2SSFC, and TYPE-3SSFC with and without silica fume in the 5% sulfuric acid solution at the durations of 28, 56, 91, and 182 days, the coefficient of determination was estimated as $R^{2}\;=\;0.7206$, $R^{2}\;=\;0.8365$, $R^{2}\;=\;0.9291,$ and $R^{2}\;=\;0.9919,$ respectively. Figure 7 also suggests that the correlation coefficient of mass loss regarding compressive strength reduction is high with time because compressive strength reduction decreases with an increase in time in a (5% H_2_SO_4_ solution). It also shows that compressive strength reduction decreases with the addition of silica fume. It can be seen from Figure 7 that the $R^{2}\;$value increased as the duration of the samples increased in a (5% H_2_SO_4_ solution). The highest value was reported for a 182-day immersion period: $R^{2}\;=\;0.9919$. As and when the duration was increased beyond 28 days, the variation in pH was reported, and the neutralization reaction slowed down. A slight variation in mass increment was observed 56 and 91 days later. This phenomenon was about to be neutralized when the immersion period reached 182 days, and a closed relationship was seen, as shown in Figure 7.

## 4. Morphology of Sustainable Concrete Made by the Different Sand Combinations

**Figure 8:**
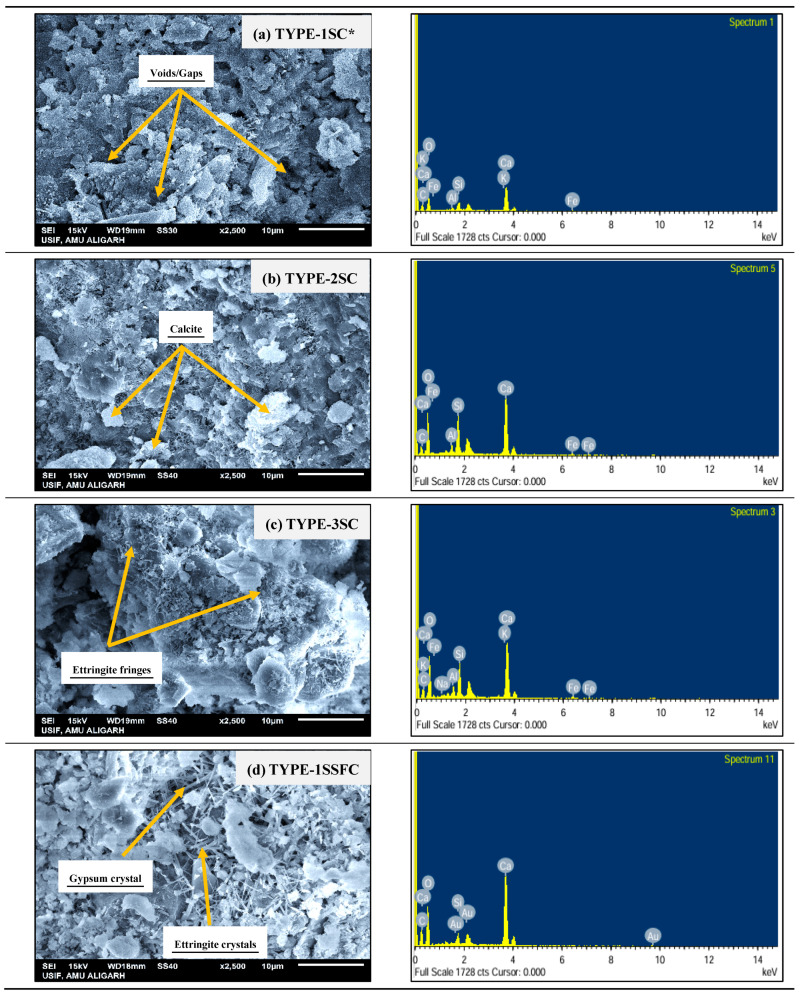

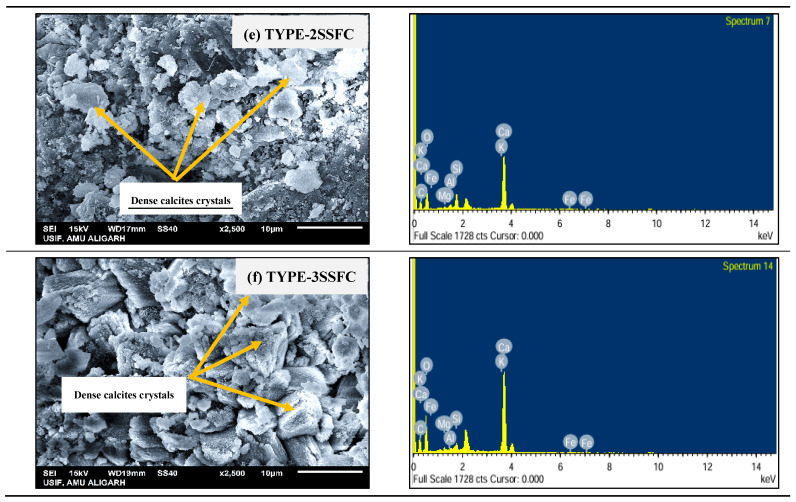
(**a**–**f**) SEM-EDS of developed concrete mixes with and without silica fume.

The structure of the particle behavior of the prepared mixes TYPE-1SC*, TYPE-2SC, TYPE-3SC, TYPE-1SSFC, TYPE-2SSFC, and TYPE-3SSFC is shown in Figure 8a–f. SEM instrumental analysis was utilized to understand the structural behavior of the prepared mixes. A 5 mm fine powder for each tested mix was used for analysis. The SEM examines a more detailed, enlarged visual depiction of the matrix. The most crucial characterization in concrete mixtures is to search for CSH gel formation, a critical strength formation in the hardened concrete structure. With instrumental SEM analysis, EDS is also employed for elemental analysis of the samples, as shown in Figure 8a–f.

TYPE-1SC* with (100% manufactured sand) showed regular voids and some patches of loose CSH and calcite in the mix. In the mix, TYPE-2SC (100% recycled sand) identified the calcite, CSH gel, and traces of ettringite. TYPE-3SC (50% recycled sand + 45% desert sand + 5% crumb rubber) showed calcite crystal, ettringite grouping, and dense CSH formation. The mix TYPE-3SC with crumb rubber content showed denser calcite formation than those without crumb rubber. The crumb rubber showed hydroxyl, carbonyl, and sulfonate groups on the surface modification. This surface modification can enhance the interface response between cement matrix materials and crumb rubbers by making the rubber surface hydrophilic and drastically lowering its contact angle with water.

Furthermore, the bond developed between aggregate and SCMs was strengthened by the mineralization process activated by microsilica. In the mix, TYPE-1SSFC with 10% optimized silica fume reported the gypsum mass along with the ettringite rod and calcite crystal. TYPE-2SSFC (100% recycled sand) and 10% optimized silica fume appear to cause dense CSH and calcite formation in the mix. This is because the leftover cement in recycled aggregate further reacts with silica fume. For the mix, TYPE-3SSFC with (50% recycled sand + 45% desert sand + 5% crumb rubber) and 10% silica fume content showed that the interface improved due to the dense interconnectivity of the concrete mix. This is due to the interface between the paste’s crumb rubber and silica fume content. A dense calcite crystal was also seen in the mixture.

The EDS attached with SEM provides the elemental composition of the prepared mixes, as shown in Figure 8a–f. The sand with crumb rubber comprises O, Ca, Si, and C, and a minor fraction of Al, Fe, K, and Na, respectively. Furthermore, silica fume in the mixes comprises the main components O, Ca, C, and Au, with a small quantity of Si with a lesser magnitude of Al, Mg, Fe, and K. Also found was a higher magnitude of the main components in terms of O, Ca, and C with the obtaining of the lower range of Si, Mg, Fe, and Al. The mixes TYPE-1SC*, TYPE-2SC, and TYPE-3SC were found to be 23.63%, 21.16%, and 22.07% weight percent, demonstrating that recycled fine aggregate, desert sand, and crumb rubber retain the availability of the calcium source. This concrete within microsilica shows better calcium activity of 25.63%, 30.72%, and 31.05% in the mixes TYPE-1SSFC, TYPE-2SSFC, and TYPE-3SSFC. The increased Ca showed unhydrated cementitious elements, mostly leftover cement from recycled sand and freshly added silica fume content.

**Figure 9:**
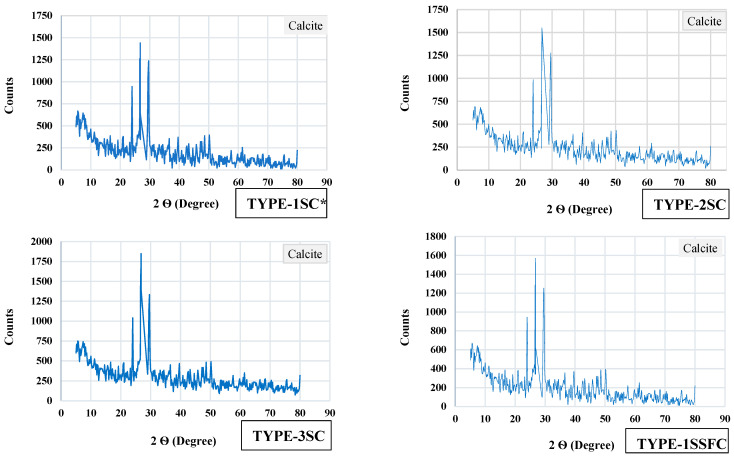

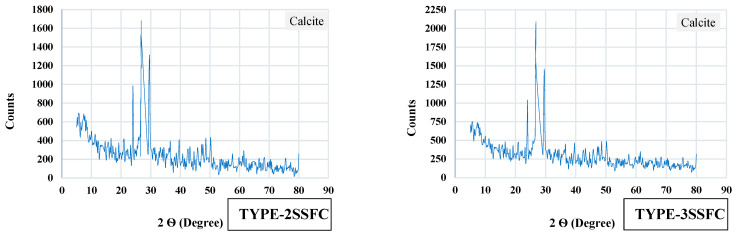
XRD behavior of developed concrete mixes with and without silica fume.

The XRD-6100 X-ray diffractometer instrument (Shimadzu, Kyoto, Japan) was utilized to examine the X-ray (XRD) and investigate the composition of the complete depth mixes. The samples were crushed into a fine powder to perform an XRD test. The 2θ range in the angle of (5–80°) was assimilated. All the prepared mixes, TYPE-1SC*, TYPE-2SC, TYPE-3SC, TYPE-1SSFC, TYPE-2SSFC, and TYPE-3SSFC, are presented in Figure 9. In the calcite crystal between the angles of (5–80°) of 2θ, numerous peaks were observed. A strong peak was reported at 26° of 2θ. The XRD analysis validated the CaCO_3_ calcite form, and the pozzolanic activity was consistent [53,54]. The mix TYPE-3SSFC with the combination of (50% recycled sand + 45% desert sand + 5% crumb rubber) and 10% silica fume content showed an intense peak of calcite in the XRD spectra compared to the other mixes. When the microstructure of the concrete mixes was improved, the concrete matrix became more resilient due to the higher calcite content. Moreover, higher calcite levels were associated with stronger concrete matrix interconnectivity and higher recovery outcomes regarding compressive strength and temperature resistance. This phenomenon is more active when silica fume is added to the newly developed sand types. It has also been observed that the TYPE 2-Sand (100% recycled sand) with 10% optimized silica content added increases the hydration process because extra leftover layers of recycled sand help increase the hydration and produce secondary CSH gel in the mixes. Furthermore, adding the crumb rubber content provides more dense interconnectivity of the concrete mix. This is due to the interface between crumb rubber and the silica fume content of the paste.

## 5. Limitations and Prospects of the Study

There are several limitations of this study. First of all, locally available recycled sand and desert sand were utilized in this study. Using desert sand from other countries may affect its physical and chemical properties. No standard methodology is available for the preparation and utilization of recycled concrete. For this reason, different studies showed variations in results even with the same replacement ratios. That could alter the findings of this study. In addition, standard requirements for measuring the durability of prepared concrete at elevated temperatures and under sulfuric acid solution are not available. All studies are based on past judgments of the published studies. It would affect the validation of results.

The study aimed to replace 100% natural sand with a newly developed sustainable sand combination (50% recycled sand + 45% desert sand + 5% crumb rubber) to provide sustainable sand for the concrete industry. The durability of the prepared mixes was evaluated at high temperatures of (150–750 °C) and against immersion in a 5% sulfuric acid solution for 28, 56, 91, and 182 days, which was the objective of this study. The experimental study was conducted to report the compressive strength, residual compressive strength, mass loss, and the morphological characteristics of the developed mixes. As we know, each study opens a new research direction in the same area. However, extensive research is needed to evaluate the durability of sustainable concrete developed in this study for the construction industry. Some more experimental data can be generated by performing the new tests, such as the effects of the high temperatures and sulfuric acid solution on the modulus of elasticity of developed sand concrete. Corrosion and freeze–thaw cycle tests can be performed on the developed sand concrete used in this study in future work.

## 6. Conclusions

A laboratory study evaluated the performance of sustainable sand concrete according to the durability characteristics under elevated temperature and against sulfuric acid solution. For this study, three concrete mixes were prepared with three newly developed sand types: TYPE 1-Sand with (100% manufactured sand), TYPE 2-Sand (100% recycled sand), and TYPE 3-Sand with the combination (50% recycled sand + 45% desert sand + 5% crumb rubber), respectively. Three additional mixes were also prepared by adding optimized 10% silica fume content to each mix. The durability of these developed mixes was evaluated by exposing the samples of prepared mixes to high temperatures (150–750 °C). The durability against (5% H_2_SO_4_ solution) was also measured at 28, 56, 91, and 182 days of immersion. The main conclusions of this study are listed pointwise, as follows.

1. It is concluded from the results of the study that newly developed sand concrete TYPE-1SSFC, TYPE-2SSFC, and TYPE-3SSFC with the optimized 10% silica fume showed less deterioration in the samples at each heating stage (150, 300, 450, 600, and 750 °C) compared to the mixes TYPE-1SC*, TYPE-2SC, and TYPE-3SC without silica fume content.
2. The sustainable mix TYPE-3SSFC with the sand combination (50% recycled sand + 45% desert sand + 5% crumb rubber) with 10% optimized silica fume content revealed more resistance than the other mixes at high temperatures. The compressive strength in the mix TYPE-3SSFC was 20.6%, 16.3%, 14.7%, 21.3%, 26.5%, and 43.2% higher than the mix TYPE-3SC without 10% silica fume at (150, 300, 450, 600, and 750 °C), respectively.
3. The mass loss in the developed mix TYPE-3SSFC with 10% optimized silica fume after 28, 56, 91, and 182 days in (5% H_2_SO_4_ solution) was found (4.19, 4.38, 4.16, and 4.08) and showed better performance against the sulfuric acid solution compared to other mixes. However, the pattern of the results was the same in all mixes: the maximum deterioration of samples against a (5% H_2_SO_4_ solution) was seen within the first 28 days of immersion, and then the rate of deterioration was slowed. Secondly, the deterioration was neutralized at 182 days of immersion and did not show a noticeable reduction. Finally, it was also concluded that the mix TYPE-3SSFC with 10% silica fume has a better resistance against 5% sulfuric acid solution than the mix without silica fume.
4. A linear correlation coefficient between mass loss and compressive strength reduction was obtained during the immersion of samples in 5% H_2_SO_4_ solution. It can be found from the results that the $R^{2}\;$value increased as the duration of the samples increased, and the highest value was reported following 182 days of immersion period: $R^{2}\;=\;0.9919$. As and when the duration was increased beyond 28 days, the variation in pH was reported, and the neutralization reaction slowed down. A slight variation in mass increment was observed at 56 and 91 days. This phenomenon would be neutralized when the immersion period reached about 182 days.
5. The microstructural investigation (SEM-EDS) in the prepared concrete mix TYPE-3SSFC combination shows the establishment of CSH gel and calcite crystals, which solidify into a solid mass, creating a more compact and solid matrix. This was due to the availability of calcite precipitations in recycled sand and silica fume in the mix. Hence, it is concluded that it was crucial to stabilize the solid matrix structure, which ensured better stability against high thermal resistance and acid attacks.
6. The mix with the sustainable TYPE 3-Sand combination (50% recycled sand + 45% desert sand + 5% crumb rubber) with 10% optimized silica fume performed almost the same as the reference mix with all natural materials. The mix with TYPE 2-Sand (100% recycled sand) revealed inferior results, low stability, and high damage. Finally, it was concluded that the developed TYPE 3-Sand with optimized 10% silica fume content showed better resistance against high temperatures and (5% H_2_SO_4_ solution).

## Figures and Tables

**Table 1:** Combination of prepared sand TYPES 1, 2, and 3.

Sand Designation	Manufactured and Recycled Sand			Desert Sand	Crumb Rubber	Total
	Fraction			Fraction	Fraction	
	(4.75–2.36) mm	(2.36–1.18) mm	(1.18–0.075) mm	(2.36–0.30) mm	(1.18–0.150) mm	
TYPE 1-Sand	20%	40%	40%	-	-	100%
TYPE 2-Sand	20%	40%	40%	-	-	100%
TYPE 3-Sand	20%	15%	15%	45%	5%	100%

**Table 2:** Physical parameters of the concrete mix design of this study.

Physical Parameters	Values	Standard
Specified compressive strength (*f′_c_*)	35 MPa	[36,37]
Required average compressive strength (*f′_cr_*)	43 MPa	[36,37]
Required Slump	75–100 mm	[36]
Maximum size of aggregate	19 mm	[36]
Fineness Modulus (FM) of TYPE 1-Sand	2.8	[36]
Fineness Modulus (FM) of TYPE 2-Sand	2.7	[36]
Fineness Modulus (FM) of TYPE 3-Sand	2.6	[36]
Grading of aggregate as satisfactory	Within upper and lower limits	[37,38]
The bulk specific gravity of natural coarse aggregate	2.920	[39]
The bulk specific gravity of TYPE 1-Sand	2.808	[40]
The bulk specific gravity of TYPE 2-Sand	2.634	[40]
The bulk specific gravity of TYPE 3-Sand	2.746	[40]
Sand equivalent value of TYPE 1-Sand	95%	[41]
Sand equivalent value of TYPE 2-Sand	92%	[41]
Sand equivalent value of TYPE 3-Sand	89%	[41]
Rodded bulk density of coarse aggregate	1598 kg/m^3^	[42]
Absorption capacity of coarse aggregate	0.88	[39]
Absorption capacity of TYPE 1-Sand	0.90	[40]
Absorption capacity of TYPE 2-Sand	6.429	[40]
Absorption capacity of TYPE 3-Sand	3.806	[40]
Moisture content of fine and coarse aggregate	Zero	[36]
Exposure conditions	Normal	[36]

**Table 3:** Concrete component proportions.

Mix Designation	Concrete Component Proportions										
	Cementitious Materials		Combination of Fine Aggregates				Natural Coarse Aggregate	OPC kg/m^3^	Water kg/m^3^	Admixture by Weight of Cement (%)	Slump mm
	OPC (%)	SF (%)	MS (%)	RS (%)	DS (%)	CR (%)	NCA %				
TYPE-1SC*	100		100	-	-	-	100	436	205	0.8	100
TYPE-2SC	100			100	-	-	100	436	205	1.8	100
TYPE-3SC	100			50	45	5	100	436	205	1.3	100
TYPE-1SSFC	90	10	100	-	-	-	100	392.4	205	1.1	100
TYPE-2SSFC	90	10		100	-	-	100	392.4	205	2.2	100
TYPE-3SSFC	90	10		50	45	5	100	392.4	205	1.6	100

**Table 4:** Program operating input data.

Temperature and Time Input	Input Data in the Program for 150 °C	Input Data in the Program for 300 °C	Input Data in the Program for 450 °C	Input Data in the Program for 600 °C	Input Data in the Program for 750 °C	Relevance to the Program
T-01	0	0	0	0	0	Initial temperature input
t-01	30	60	90	120	150	Increasing the temperature at a rate of heating by 5 degrees per minute
T-02	150	300	450	600	750	Max temperature heating stage
t-02	120	120	120	120	120	Temperature flat 120 min at max temperature heating stage
T-03	150	300	450	600	750	Max temperature heating stage
t-03	30	60	90	120	150	Decreasing the temperature with an average heating of 5 °C/min
T-04	0	0	0	0	0	Initial temperature reached
t-04	−121	−121	−121	−121	−121	end of program

## Data Availability

The original contributions presented in this study are included in the article. Further inquiries can be directed to the corresponding author.
